# Periodontitis and Sjogren’s syndrome: a bidirectional two-sample mendelian randomization study

**DOI:** 10.1186/s12903-024-04151-7

**Published:** 2024-03-25

**Authors:** Yixuan Liu, Nuozhou Liu, Peiyan Sun, Yi Liu, Wei Hua

**Affiliations:** 1 State Key Laboratory of Oral Diseases, West China Hospital of Stomatology, National Clinical Research Center for Oral Diseases, Sichuan University, Chengdu, 610041 China; 2 West China School of Medicine, West China Hospital, Sichuan University, Chengdu, 610041 China; 3 Department of Rheumatology and Immunology, West China Hospital, Sichuan University, Chengdu, 610041 China; 4 Department of Dermatovenereology, West China Hospital, Sichuan University, Chengdu, 610041 China; 5 Cosmetic Safety and Efficacy Evaluation Center of West China Hospital, Sichuan University, Chengdu, 610041 China

**Keywords:** Periodontitis, Sjogren’s syndrome, Mendelian randomization, Causality, Epidemiology

## Abstract

**Objectives:**

Observational studies indicated a controversial relationship between periodontitis (PD) and Sjogren’s syndrome (SS). To overcome restrictions in conventional observational studies, we conducted a two-sample Mendelian randomization (MR) analysis to assess the potential bidirectional relationship between PD and SS.

**Methods:**

We utilized the largest available genome-wide association study (GWAS) of European ancestry on both PD (17,353 cases-28,210 controls) and SS (2495 cases-365,533 controls) for MR genetic instrument selection. The random-effect inverse-variance weighted (IVW) method complemented by Causal Analysis Using Summary Effect (CAUSE), weighted median, weighted mode, simple mode, MR-Egger regression, and MR-pleiotropy residual sum and outlier (MR-PRESSO) was used for MR analysis. Subsequent pleiotropy and heterogeneity tests were conducted.

**Results:**

IVW analysis exhibited neither an effect of PD on SS (OR = 0.939, 95%CI = 0.525–1.677, *P* = 0.8304) nor that of SS on PD (OR = 1.007, 95%CI = 0.977–1.038, *P* = 0.6440). The other five complementary methods further recognized the null association with an effect size close to one. No significant pleiotropy was detected in the relationship between PD and SS (*P* > 0.05). Heterogeneity existed in the effect of PD on SS but not vice versa.

**Conclusions:**

No genetic causality between PD and SS or vice versa was supported by our results under MR assumptions and limitations. The study results provided new insights into the relationship between periodontal status and sjogren’s syndrome, highlighting the need for a more prudent medical intervention.

**Supplementary Information:**

The online version contains supplementary material available at 10.1186/s12903-024-04151-7.

## Introduction

Periodontitis (PD) is a common dysbiotic inflammatory disorder indicated by impaired integrity of periodontium, including alveolar bone, cementum, gingiva, and periodontal ligament [1–3]. The continuous breakdown of periodontium tissue eventually leads to tooth looseness and the loss of teeth if not properly treated, which severely affects patients’ quality of life and causes enormous socioeconomic burden [4, 5]. About 20–50% of the global population suffer from PD, while severe PD affects approximately 15% of the general population, making it a leading prevalent chronic inflammatory disease worldwide [6, 7]. Notably, though PD has a complex etiology at multiple levels, such as dysbiotic microbes followed by destructive inflammation, previous studies have shown that genetic susceptibility greatly contributes to the etiopathogenesis of PD [1, 8].

Sjogren’s syndrome (SS) is an autoimmune disease with unspecified etiology which primarily involves the lacrimal and salivary glands, leading to excessive xerophthalmia (dry eyes) and xerostomia (dry mouth) [9]. Moreover, SS patients can develop many systemic manifestations due to autoimmune-mediated involvement in multiple organs, such as immune thrombocytopenia, interstitial lung disease, and cardiovascular disease [10, 11]. Systemic sicca symptoms that can only be relieved by symptomatic treatments and possibly associated fatigue, depression, and decreased physical performance severely jeopardize patients’ quality of life and increase their financial burden [12].

It has long been recognized that PD might be linked with multiple comorbidities, like cardiovascular disease, chronic kidney disease, and diabetes [13, 14]. In terms of PD-related autoimmune diseases, a bidirectional relationship between PD and rheumatoid arthritis was supported by many epidemiological studies [15]. However, as for SS, this link remains controversial. Several epidemiological studies highlighted that SS patients had a higher risk of developing PD [16, 17], while patients with PD were also more susceptible to subsequent SS development [18]. Meanwhile, some key proinflammatory mediators that might drive a higher risk of PD are elevated in saliva extracted from patients with SS, such as IL-12, IL-6, and IL-17, indicating the possible existence of a shared etiopathogenesis among these two diseases [19–22]. Several recent meta-analyses also supported these findings [23–25]. However, there were also some studies reporting no link between PD and SS [26, 27].

To overcome the restriction and bias of conventional observational studies, including but restricted to measurement error, reverse causality, and residual confounding, an alternative method called Mendelian randomization (MR) has a huge application value in exploring the relationship between PD and SS. MR utilizes exposure/outcome–associated single nucleotide polymorphisms (SNPs) as instrumental variables (IVs) that are distributed during meiosis at random and not subject to reverse causation or residual confounding [28, 29]. Hence, a bidirectional two-sample MR study was conducted to assess the possible causal association between PD and SS or vice versa by using statistics from the largest genome-wide association studies (GWAS) of PD from the GLIDE consortium and SS from FinnGen consortiums [30, 31].

## Materials and methods

### Study design

A bidirectional two-sample MR was carried out to assess the possible causal association between PD and SS. IVs randomly allocated during meiosis provides a good opportunity to overcome reverse causation or residual confounding in conventional observational studies [28, 29]. Three key assumptions of IVs must be simultaneously satisfied to gain reliable MR results: (1) relevance: the genetic IVs are strongly correlated with the exposure; (2) exchangeability: the genetic IVs have no link to any confounders affecting the chosen exposure and outcome; (3) exclusion restriction: the genetic IVs influence outcome solely via the exposure [32].

All data analysis was operated in R (version 4.1.3) through the package TwoSampleMR (0.5.6), cause (1.2.0) and MRPRESSO (1.0). This paper followed the STROBE-MR guideline [33]. In addition, all primary data were derived from the largest publicly accessible GWAS, and no independent ethical approval was necessary.

### Data sources

The largest available GWAS on both PD and SS were utilized to obtain proper genetic instruments in our two-sample MR analysis [30, 31]. Twelve independent study contributed to GWAS on PD with 17,353 PD cases and 28,210 controls, including (1) ARIC-Atherosclerosis Risk in Communities; (2) COHRA1-The Center for Oral Health in Appalachia cohort 1; (3) DRDR-the Dental Registry and DNA Repository of the University of Pittsburgh School of Dental Medicine; (4) HCHS/SOL-the Hispanic Community Health Study/Study of Latinos; (5) MDC-the Malmö Diet and Cancer Study; (6) NFBC 1966-the Northern Finland Birth Cohort 1966; (7) SHIP-the Study of Health in Pomerania; (8) SHIP Trend-the Study of Health in Pomerania Trend; (9) TWINGENE-a genotyped epidemiological study recruited from the Swedish Twin Registry; (10) WGHS-the Women’s Genome Health Study; (11) BBJ-Biobank Japan and (12) TMDUAGP-Tokyo Medical and Dental University Aggressive Periodontitis Study [30]. Participants reached the criteria of the Community Periodontal Index (CPI), Centers for Disease Control and Prevention (CDC)/American Academy of Periodontology (AAP) definition, or self-reports of PD diagnosis were considered as PD cases [34].

The summary statistics for the SS dataset were based on FinnGen Release 9, managed by the University of Helsinki [31]. A total of 2495 clinically diagnosed cases and 365,533 controls were accessed via phenocode ‘M13_SJOGREN’. M35.0 (ICD-10), 7102 (ICD-9), and 73,490 (ICD-8) were included to diagnose SS patients, mainly by ICD-10 code. Both GWAS were carried out in individuals of European Caucasian ancestry with approvement from the local institutional review board and ethics committee [30, 31]. The data description was also summarized in Table 1.

**Table 1:** Description of GWAS used for each phenotype

Phenotype	Data sources	Cases	Controls	Sample size	SNPs(n)	Ancestry
PD	GLIDE consortium	17,353	28,210	45,563	396,701	European
SS	FinnGen	2495	365,533	368,028	20,170,011	European

GWAS, Genome-wide association studies; PD, Periodontitis; SS, Sjogren’s syndrome; SNPs, Single nucleotide polymorphisms.

### Selection of the genetic instruments

We exploited a series of standard control strategies to find valid IVs fulfilling three core assumptions of MR. Initially, we selected SNPs for PD that were randomly allocated and reached a strict threshold (*P* < 5*10^− 8^), while a more lenient level of *P* < 5*10^− 5^ was applied to encompassing enough SNPs for SS. The clumping procedure (r^2^ < 0.001 and clumping size = 10,000 kb) was conducted to account for linkage disequilibrium (LD) and thus guarantee independence among SNPs [35]. We excluded SNPs harboring a direct association with the outcome (*P* < 5*10^–8^) to reach the third MR assumption. Furthermore, we searched for potential risk confounders in PhenoScanner (www.phenoscanner.medschl.cam.ac.uk/) to assure that the genetic IVs affect outcome solely via exposure.

To reduce possible weak IV bias, F-statistic for each IV was computed via the following formula: F = R^2^×(N-2)/(1-R^2^). R^2^ represents the percentage of explained variance of the IVs, and F-statistics ≥ 10 were recognized as strong IVs [36]. Finally, we harmonized the data to ensure that the effect sizes of exposures and outcomes aligned to identical effect alleles using the R command “harmonize_data”.

### Mendelian randomization analysis

A variety of MR approaches were used to explore the possible causal association between PD and SS. The major MR outcome was based on random-effect the inverse variance weighted (IVW) method, complemented by simple mode, weighted mode, MR‒Egger regression, and the weighted median to demonstrate causal correlations [37]. The statistical power was computed through the mRnd website (https://shiny.cnsgenomics.com/mRnd/) [38].

To assess and address possible pleiotropy, MR‒Egger regression with an intercept test was carried out, where P value ≤ 0.05 was recognized as significant horizontal pleiotropy [39]. Pleiotropy-robust methods, including the MR Pleiotropy REsidual Sum and Outlier (MR-PRESSO) and weighted median, were performed to account for possible pleiotropy [40, 41]. However, these two complementary methods tend to provide wider confidence intervals (CIs), thus exhibiting lower precision than IVW [42]. In addition, we added Causal Analysis Using Summary Effect estimates (CAUSE) approach to validate our MR analysis, which could theoretically classify causality from correlated pleiotropy (a compromise of the second MR assumption, arising when variants affect both exposure and outcome through a shared genetic factor) [43], consider possible uncorrelated horizontal pleiotropy (a compromise of the third MR assumption), and minimize false positives induced by shared heritable factors in other MR approaches [44]. To examine heterogeneity, Cochran’s Q statistic was calculated, where a random-effect model was conducted when the P value ≤ 0.05 [45]. A funnel plot was used to examine polymorphisms and thus gauge the reliability of the study results. Leave-one-out analysis eliminating one SNP at a time was conducted to diminish bias from a single SNP and thus detect the robustness and consistency of our results.

## Results

### Causal effects of PD on SS

**Fig. 1:**
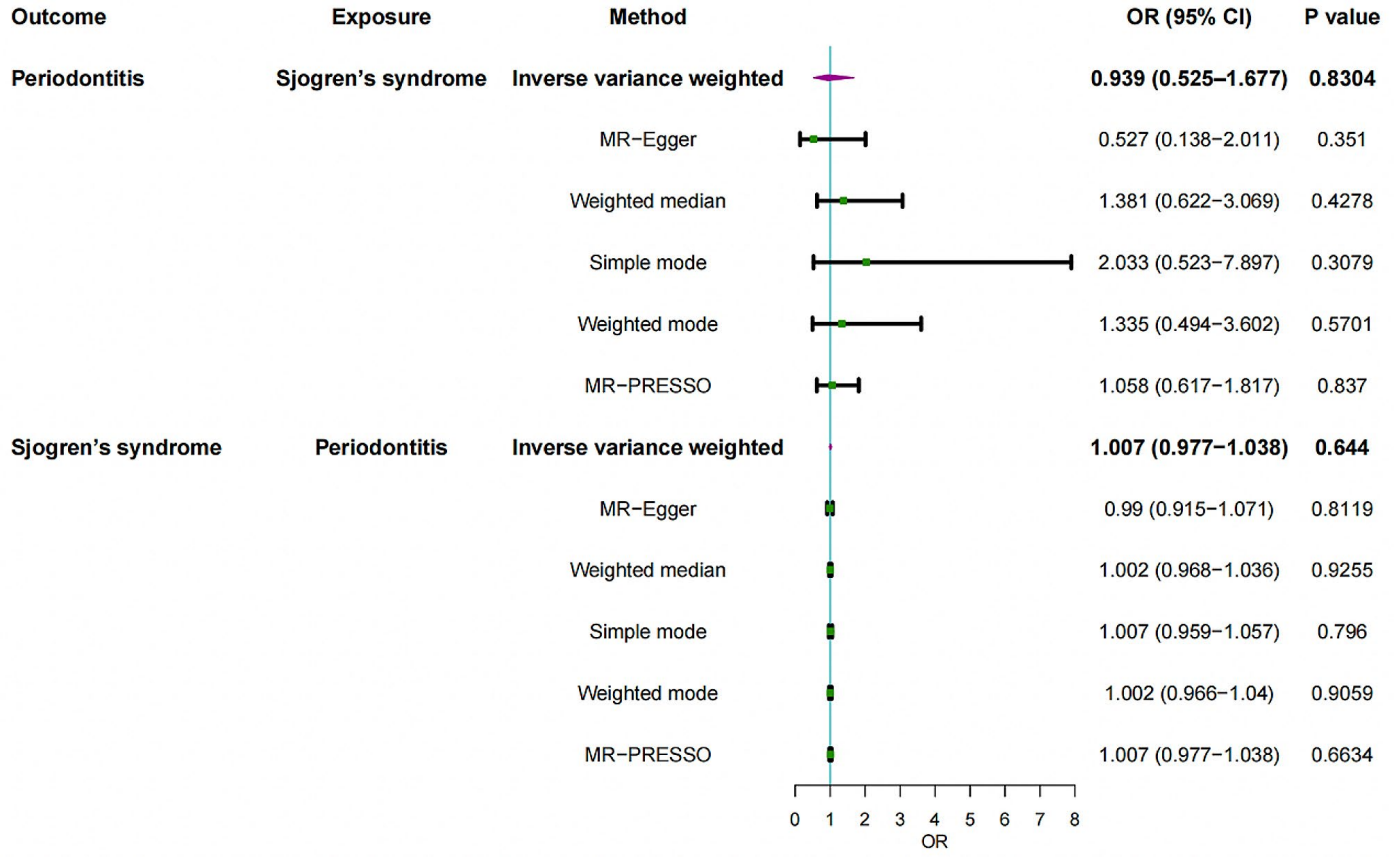
Mendelian randomization estimates for the relationship between genetically instrumented periodontitis and Sjögren’s syndrome, and vice versa. OR, odds ratio; CI, confidence interval; IVW, inverse-variance weighted; MR-PRESSO, MR Pleiotropy RESidual Sum and Outlier

**Fig. 2:**
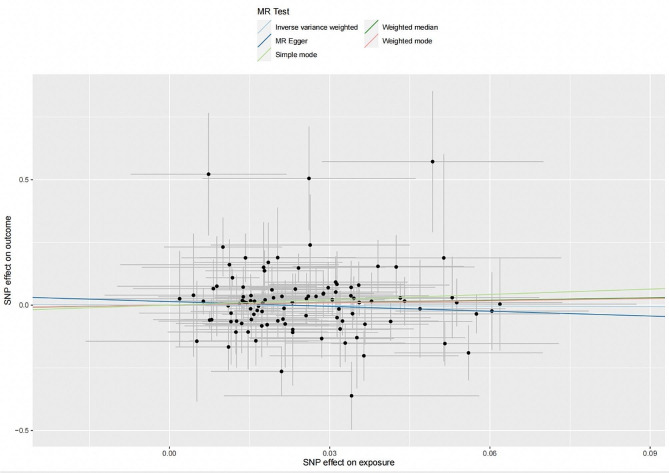
Scatterplot of the genetic association between periodontitis and Sjögren’s syndrome. Genetic association of periodontitis with Sjögren’s syndrome

104 SNPs in total were finally selected as IVs to investigate the possible causal impact of PD on SS, where their associated descriptive statistics were summarized in Table S1. 12.5% of the total variance was explained by these SNPs extracted for PD as exposure, with an average F-statistic of 77.1. No significant causal impact of PD on SS was detected through the IVW method (OR = 0.939, 95%CI = 0.525–1.677, *P* = 0.8304) followed by other four conventional MR methods (Fig. 1). MR-PRESSO (OR = 1.058, 95%CI = 0.617–1.817, *P* = 0.8370) also corroborated these results, with no outlier detected. The CAUSE analysis also showed no significant impact of PD on SS (OR = 1.51, 95% CI = 0.01–259.82, *P* = 0.93). No significant horizontal pleiotropy was detected by the MR‒Egger intercept test with scatterplot (intercept = 0.0140; *P* = 0.3511) **(**Fig. 2**and** Table 2**)**. Heterogeneity was identified through Cochran’s Q (*P* = 0.0419 for IVW; *P* = 0.0418 for MR‒Egger) (Table 2), while no high-influence leverage SNP was exhibited in leave-one-out analysis (Figure S1). In addition, the funnel plot was symmetrical (Figure S3).

**Table 2:** Heterogeneity test and pleiotropy test of genetic variants

Exposure		Heterogeneity				Pleiotropy	
	Outcome	MR Egger		IVW		MR Egger	
		Cochran’s Q	P value	Cochran’s Q	P value	Egger intercept	P value
Periodontitis	Sjogren’s syndrome	127.9803	0.0418	129.0815	0.0419	0.0140	0.3511
Sjogren’s syndrome	Periodontitis	7.8305	0.0980	8.2702	0.1420	0.0037	0.6602

Q, heterogeneity statistic Q

### Causal effect of SS on PD

**Fig. 3:**
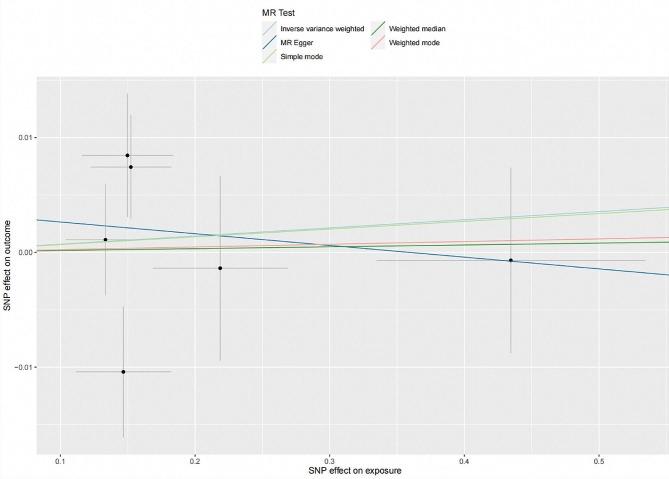
Scatterplot of the genetic association between Sjögren’s syndrome and periodontitis. Genetic association of Sjögren’s syndrome with periodontitis

Six SNPs explaining 5.01% of the variance of SS were extracted as exposure for SS (**Table S2**). The average F-statistic was 3099.6. Similarly, we found no significant causal impact of SS on PD by IVW method (OR = 1.007, 95%CI = 0.977–1.038, *P* = 0.6440) complemented by four other MR methods. MR-PRESSO did not find any outliers (OR = 1.007, 95%CI = 0.977–1.038, *P* = 0.6634). CAUSE indicated no causal effect for SS on PD (OR = 0.852, 95% CI = 0.01–90.92, *P* = 0.86). Neither heterogeneity between individual SNPs nor horizontal pleiotropy was found according to Cochran’s Q (*P* = 0.6602 for IVW; *P* = 0.0418 for MR‒Egger) and MR‒Egger intercept test with scatterplot, respectively (intercept = 0.0037; *P* = 0.1420) (Fig. 3). Leave-one-out analysis indicated a robust result where there was no single SNP strongly affecting the overall causal estimation (Figure S2). Due to the small number of SNPs for SS, the interpretation of funnel plot (Figure S4) should be cautious.

## Discussion

Up to now, this is the first bidirectional two-sample MR study based on the largest available GWAS to explore the potential causal association between PD and SS. No causal link between PD and SS or vice versa was found on the basis of mendelian randomization assumptions.

Previous researches assessing the relationship between PD and SS seemed very contradictory. Our findings are in line with several previous studies that found no significant association between PD and SS, while those studies solely focused on the possible risk of PD or unfavorable periodontal status among patients with SS without considering whether PD patients have a different likelihood of developing SS versus the general population [26, 27]. However, there were also several studies in contrast with our results. Two systematic reviews with meta-analysis similarly reported that SS patients were more likely to suffer from PD or worse periodontal status, which was not in line with our results [23, 25]. However, these two studies still did not examine the risk of developing SS among PD patients, and as kindly figured out by their authors, the reliability of these results was impaired by unsatisfied methodological quality of the primary studies in meta-analysis, high statistical heterogeneity with no explicit source, or selection and cofounding bias in observational studies. Only two studies considered that PD patients might have a different likelihood of developing newly diagnosed SS comparing to the general population, both of which reported that PD patients had higher risk of developing SS [18, 46]. Another systematic review without meta-analysis further recognized that current evidence about the association between PD and SS or vice versa was still inconclusive [47]. Possible explanations for the discordance among these observational studies included but are not restricted to potential confounders, such as smoking and other environmental factors, different population settings, and misdiagnosis of early-stage SS with only minor clinical manifestations. These shortcomings in conventional observational studies could lead to significant heterogeneity [48].

Our study possesses multiple strengths. Initially, this is the first MR research derived from the largest GWAS on both PD and SS currently available, complying with the STROBE-MR statement [33]. Prudential MR design with the IVW method complemented by four traditional MR methods, CAUSE analysis, and sensitivity analyses has great potential in avoiding reverse causality and residual confounding in traditional observational studies. In addition, there was no evidence for any IV of both PD and SS being correlated to potential confounding factors by searching the PhenoScanner. The IVs in this study are strong enough (F statistic much greater than 10) to diminish potential bias from sample overlap.

Although the MR design gives us a valuable opportunity to explore possible causality between PD and SS, there are still some limitations. Although we tried to extract genetically similar cohorts, the genetical difference between GLIDE consortium on European populations and FinnGen on Finnish samples could introduce bias in our study. However, the GLIDE consortium on the European comprises a subset of participants of European Caucasian ancestry from Finland, which partially intersected with participants from FinnGen database for Sjogren’s syndrome and reduced bias. The subsequent heterogeneity could be further improved by valid measures of the oral disease or endpoint and IVW method with random effects as the major analysis [49, 50]. Moreover, both two GWAS on PD and SS were derived from individuals of European ancestry, which might cause potential overlap in the exposure and outcome cohorts and hinder the generalizability of our study. To encompass enough SNPs for SS as IV, a more lenient threshold (*P* < 5*10^− 5^) was applied [51]. A total of 104 SNPs were extracted for PD as exposure and expressed 12.5% of the variance, while only 6 SNPs were selected for SS as exposure, explaining only 5.01% of the variance. Since 5 of 6 SNPs for SS had low effect allele frequency (< 0.05) (**Table S2**), we have to reserve these SNPs instead of deletion. Although a satisfied statistic power (100% to yield an OR of 1.20) was established to explain an impact of PD on SS, a lower power existed in the effect of SS on PD (13% power to yield an OR of 1.20). Inherent heterogeneity was found with the effect of PD on SS, which is likely to influence results. Fortunately, it could be accepted since we utilized IVW method with random effects as the major analysis [32]. Regarding the interpretation and future practice of our MR analysis, its evidence quality is below that of standard randomized clinical trials (RCTs) and systematic reviews of RCTs according to the evidence-based pyramid [43, 52]. Last, deidentified GWAS data without specific personal information impeded us from performing a more detailed subgroup analysis based on more specific population settings, where future studies can be improved as the GWAS database expands and is refined.

## Conclusions

No genetic causality between PD and SS or vice versa was supported by our results under MR assumptions and limitations. The study results provided new insights into the correlation between periodontal status and SS, highlighting the need for a more prudent medical intervention.

### Electronic supplementary material

Below is the link to the electronic supplementary material.

Supplementary Material 1

## Data Availability

The GWAS statistics for periodontitis and Sjögren’s syndrome are publicly accessible at https://data.bris.ac.uk/data/dataset/2j2rqgzedxlq02oqbb4vmycnc2. and the FinnGen R9 release at https://www.finngen.fi/en ,respectively.

## References

[1] Hajishengallis G (2015). Periodontitis: from microbial immune subversion to systemic inflammation. Nat Rev Immunol.

[2] Slots J. Periodontitis: facts, fallacies and the future. Periodontol 2000 2017, 75(1):7–23. 10.1111/prd.12221 28758294

[3] Kwon T, Lamster IB, Levin L (2021). Current concepts in the management of Periodontitis. Int Dent J.

[4] Peres MA, Macpherson LMD, Weyant RJ, Daly B, Venturelli R, Mathur MR, Listl S, Celeste RK, Guarnizo-Herreño CC, Kearns C (2019). Oral diseases: a global public health challenge. Lancet (London England).

[5] Kassebaum NJ, Bernabé E, Dahiya M, Bhandari B, Murray CJ, Marcenes W (2014). Global burden of severe periodontitis in 1990–2010: a systematic review and meta-regression. J Dent Res.

[6] Luo LS, Luan HH, Wu L, Shi YJ, Wang YB, Huang Q, Xie WZ, Zeng XT (2021). Secular trends in severe periodontitis incidence, prevalence and disability-adjusted life years in five Asian countries: a comparative study from 1990 to 2017. J Clin Periodontol.

[7] Kinane DF, Stathopoulou PG, Papapanou PN (2017). Periodontal diseases. Nat Reviews Disease Primers.

[8] Michalowicz BS, Diehl SR, Gunsolley JC, Sparks BS, Brooks CN, Koertge TE, Califano JV, Burmeister JA, Schenkein HA (2000). Evidence of a substantial genetic basis for risk of adult periodontitis. J Periodontol.

[9] Brito-Zerón P, Baldini C, Bootsma H, Bowman SJ, Jonsson R, Mariette X, Sivils K, Theander E, Tzioufas A, Ramos-Casals M (2016). Sjögren syndrome. Nat Reviews Disease Primers.

[10] Ramos-Casals M, Brito-Zerón P, Solans R, Camps MT, Casanovas A, Sopeña B, Díaz-López B, Rascón FJ, Qanneta R, Fraile G (2014). Systemic involvement in primary Sjogren’s syndrome evaluated by the EULAR-SS disease activity index: analysis of 921 Spanish patients (GEAS-SS Registry). Rheumatology (Oxford).

[11] Melissaropoulos K, Bogdanos D, Dimitroulas T, Sakkas LI, Kitas GD, Daoussis D (2020). Primary Sjögren’s Syndrome and Cardiovascular Disease. Curr Vasc Pharmacol.

[12] Negrini S, Emmi G, Greco M, Borro M, Sardanelli F, Murdaca G, Indiveri F, Puppo F (2022). Sjögren’s syndrome: a systemic autoimmune disease. Clin Experimental Med.

[13] Hajishengallis G, Chavakis T (2021). Local and systemic mechanisms linking periodontal disease and inflammatory comorbidities. Nat Rev Immunol.

[14] Teles F, Collman RG, Mominkhan D, Wang Y (2022). Viruses, periodontitis, and comorbidities. Periodontol 2000.

[15] Hajishengallis G (2022). Interconnection of periodontal disease and comorbidities: evidence, mechanisms, and implications. Periodontol 2000.

[16] Najera MP, al-Hashimi I, Plemons JM, F Rivera-Hidalgo, Rees TD, Haghighat N, Wright JM. Prevalence of periodontal disease in patients with Sjögren’s syndrome. Oral Surg Oral Med Oral Pathol Oral Radiol Endod. 1997;83(4):453–7. 10.1016/s1079-2104(97)90144-x 9127376

[17] Chuang CJ, Hsu CW, Lu MC, Koo M. Increased risk of developing dental diseases in patients with primary Sjögren’s syndrome-A secondary cohort analysis of population-based claims data. PLoS ONE. 2020;15(9):e0239442. 10.1371/journal.pone.0239442 PMC7500664 32946501

[18] Lin CY, Tseng CF, Liu JM, Chuang HC, Lei WT, Liu LY, Yu YC, Hsu RJ (2019). Association between Periodontal Disease and subsequent Sjögren’s syndrome: a Nationwide Population-based Cohort Study. Int J Environ Res Public Health.

[19] Melguizo-Rodríguez L, Costela-Ruiz VJ, Manzano-Moreno FJ, Ruiz C, Illescas-Montes R (2020). Salivary biomarkers and their application in the diagnosis and monitoring of the most common oral pathologies. Int J Mol Sci.

[20] Moreno-Quispe LA, Serrano J, Virto L, Sanz M, Ramírez L, Fernández-Castro M, Hernández G, López-Pintor RM (2020). Association of salivary inflammatory biomarkers with primary Sjögren’s syndrome. J oral Pathol Medicine: Official Publication Int Association Oral Pathologists Am Acad Oral Pathol.

[21] Bunte K, Beikler T (2019). Th17 cells and the IL-23/IL-17 Axis in the Pathogenesis of Periodontitis and Immune-mediated inflammatory diseases. Int J Mol Sci.

[22] Lugonja B, Yeo L, Milward MR, Smith D, Dietrich T, Chapple IL, Rauz S, Williams GP, Barone F, de Pablo P (2016). Periodontitis prevalence and serum antibody reactivity to periodontal bacteria in primary Sjögren’s syndrome: a pilot study. J Clin Periodontol.

[23] de Goés Soares L, Rocha RL, Bagordakis E, Galvão EL, Douglas-de-Oliveira DW, Falci SGM (2018). Relationship between sjögren syndrome and periodontal status: a systematic review. Oral Surg oral Med oral Pathol oral Radiol.

[24] Wu SY, Wu CY, Chen MH, Huang HY, Chen YH, Tsao YP, Lai YL, Lee SY (2021). Periodontal conditions in patients with Sjögren’s syndrome: a meta-analysis. J Dent Sci.

[25] Yang B, Pang X, Guan J, Liu X, Li X, Wang Y, Chen Z, Cheng B (2022). The association of periodontal diseases and Sjogren’s syndrome: a systematic review and meta-analysis. Front Med.

[26] Schiødt M, Christensen LB, Petersen PE, Thorn JJ (2001). Periodontal disease in primary Sjögren’s syndrome. Oral Dis.

[27] Maarse F, Jager DHJ, Alterch S, Korfage A, Forouzanfar T, Vissink A, Brand HS (2019). Sjögren’s syndrome is not a risk factor for periodontal disease: a systematic review. Clin Exp Rheumatol.

[28] Emdin CA, Khera AV, Kathiresan S (2017). Mendelian randomization. JAMA.

[29] Sekula P, Del Greco MF, Pattaro C, Köttgen A (2016). Mendelian randomization as an Approach to assess causality using Observational Data. J Am Soc Nephrology: JASN.

[30] Shungin D, Haworth S, Divaris K, Agler CS, Kamatani Y, Keun Lee M, Grinde K, Hindy G, Alaraudanjoki V, Pesonen P (2019). Genome-wide analysis of dental caries and periodontitis combining clinical and self-reported data. Nat Commun.

[31] Kurki MI, Karjalainen J, Palta P, Sipilä TP, Kristiansson K, Donner KM, Reeve MP, Laivuori H, Aavikko M, Kaunisto MA (2023). FinnGen provides genetic insights from a well-phenotyped isolated population. Nature.

[32] Burgess S, Davey Smith G, Davies NM, Dudbridge F, Gill D, Glymour MM, Hartwig FP, Holmes MV, Minelli C, Relton CL (2019). Guidelines for performing mendelian randomization investigations. Wellcome open Res.

[33] Skrivankova VW, Richmond RC, Woolf BAR, Davies NM, Swanson SA, VanderWeele TJ, Timpson NJ, Higgins JPT, Dimou N, Langenberg C (2021). Strengthening the reporting of observational studies in epidemiology using mendelian randomisation (STROBE-MR): explanation and elaboration. BMJ (Clinical Res ed).

[34] Eke PI, Page RC, Wei L, Thornton-Evans G, Genco RJ (2012). Update of the case definitions for population-based surveillance of periodontitis. J Periodontol.

[35] Abecasis GR, Altshuler D, Auton A, Brooks LD, Durbin RM, Gibbs RA, Hurles ME, McVean GA (2010). A map of human genome variation from population-scale sequencing. Nature.

[36] Burgess S, Thompson SG (2011). Avoiding bias from weak instruments in mendelian randomization studies. Int J Epidemiol.

[37] Yavorska OO, Burgess S. MendelianRandomization: an R package for performing mendelian randomization analyses using summarized data. Int J Epidemiol 2017, 46(6):1734–9. 10.1093/ije/dyx034 PMC5510723 28398548

[38] Brion MJ, Shakhbazov K, Visscher PM (2013). Calculating statistical power in mendelian randomization studies. Int J Epidemiol.

[39] Bowden J, Davey Smith G, Burgess S (2015). Mendelian randomization with invalid instruments: effect estimation and bias detection through Egger regression. Int J Epidemiol.

[40] Hartwig FP, Davey Smith G, Bowden J. Robust inference in summary data mendelian randomization via the zero modal pleiotropy assumption. Int J Epidemiol 2017, 46(6):1985–98. 10.1093/ije/dyx102 PMC5837715 29040600

[41] Verbanck M, Chen CY, Neale B, Do R (2018). Detection of widespread horizontal pleiotropy in causal relationships inferred from mendelian randomization between complex traits and diseases. Nat Genet.

[42] Ong JS, MacGregor S (2019). Implementing MR-PRESSO and GCTA-GSMR for pleiotropy assessment in mendelian randomization studies from a practitioner’s perspective. Genet Epidemiol.

[43] Baurecht H, Freuer D, Welker C, Tsoi LC, Elder JT, Ehmke B, Leitzmann MF, Holtfreter B, Baumeister SE (2022). Relationship between periodontitis and psoriasis: a two-sample mendelian randomization study. J Clin Periodontol.

[44] Morrison J, Knoblauch N, Marcus JH, Stephens M, He X (2020). Mendelian randomization accounting for correlated and uncorrelated pleiotropic effects using genome-wide summary statistics. Nat Genet.

[45] Bowden J, Hemani G, Davey Smith G (2018). Invited Commentary: detecting individual and Global Horizontal Pleiotropy in mendelian Randomization-A job for the humble heterogeneity statistic?. Am J Epidemiol.

[46] Lin TC, Tseng CF, Wang YH, Yu HC, Chang YC (2018). Patients with chronic periodontitis present increased risk for primary Sjögren syndrome: a nationwide population-based cohort study. PeerJ.

[47] Gheorghe DN, Popescu DM, Dinescu SC, Silaghi M, Surlin P, Ciurea PL (2023). Association between Sjögren’s syndrome and periodontitis: epidemiological, fundamental and clinical data: a systematic review. Diagnostics (Basel Switzerland).

[48] Richmond RC, Davey Smith G (2022). Mendelian randomization: concepts and scope. Cold Spring Harbor Perspect Med.

[49] Divaris K, Haworth S, Shaffer JR, Anttonen V, Beck JD, Furuichi Y, Holtfreter B, Jönsson D, Kocher T, Levy SM (2022). Phenotype harmonization in the GLIDE2 oral Health Genomics Consortium. J Dent Res.

[50] Burgess S, Davey Smith G, Davies NM, Dudbridge F, Gill D, Glymour MM, Hartwig FP, Kutalik Z, Holmes MV, Minelli C (2019). Guidelines for performing mendelian randomization investigations: update for summer 2023. Wellcome open Res.

[51] Yin KJ, Huang JX, Wang P, Yang XK, Tao SS, Li HM, Ni J, Pan HF (2022). No Genetic Causal Association between Periodontitis and Arthritis: a bidirectional two-sample mendelian randomization analysis. Front Immunol.

[52] Davies NM, Holmes MV, Davey Smith G (2018). Reading mendelian randomisation studies: a guide, glossary, and checklist for clinicians. BMJ (Clinical Res ed).

