# Supplementary material for: Periodontitis and Sjogren’s syndrome: a bidirectional two-sample mendelian randomization study
Source: BMC Oral Health. 2024 Mar 25;24:380. doi: 10.1186/s12903-024-04151-7 (PMC10962100; doi:10.1186/s12903-024-04151-7)

**Supplementary Material**

**Table S1 104 SNPs used as genetic instruments for the causality between periodontitis and sjogren’s syndrome**

| SNP | CHR | EA | OA | EAF | Exposure-Periodontitis | | | R^2^ | F | Outcome- Sjogren’s syndrome | | |
| --- | --- | --- | --- | --- | --- | --- | --- | --- | --- | --- | --- | --- |
|  |  |  |  |  | Beta | SE | P-value |  |  | Beta | SE | P-value |
| rs10763224 | 10 | A | G | 0.5657 | -0.01642 | 0.004287 | 0.000128 | 0.475491 | 396.2467 | 0.021284 | 0.028631 | 0.457248 |
| rs10788625 | 10 | A | G | 0.589 | 0.013099 | 0.004306 | 0.002352 | 0.49701 | 431.8995 | 0.005682 | 0.028643 | 0.842747 |
| rs10796153 | 10 | T | C | 0.1832 | 0.015799 | 0.00596 | 0.008025 | 0.308883 | 195.3528 | -0.03676 | 0.038603 | 0.340954 |
| rs10886076 | 10 | A | C | 0.9519 | -0.03427 | 0.009902 | 0.000537 | 0.085507 | 40.86929 | 0.034228 | 0.057301 | 0.550285 |
| rs11009054 | 10 | T | G | 0.0537 | 0.028835 | 0.009399 | 0.002154 | 0.107666 | 52.73862 | 0.046785 | 0.058011 | 0.419962 |
| rs111851689 | 10 | A | C | 0.0304 | -0.01351 | 0.012341 | 0.2736 | 0.05738 | 26.6072 | 0.073115 | 0.118268 | 0.536432 |
| rs11200392 | 10 | T | C | 0.2446 | -0.0179 | 0.004929 | 0.000281 | 0.356545 | 242.1992 | -0.02066 | 0.031803 | 0.516024 |
| rs112367495 | 10 | T | C | 0.0209 | 0.014722 | 0.014811 | 0.3203 | 0.042149 | 19.23402 | -0.10717 | 0.148246 | 0.469749 |
| rs11239839 | 10 | T | C | 0.0114 | 0.049263 | 0.020708 | 0.01736 | 0.024874 | 11.14965 | 0.572149 | 0.280494 | 0.041371 |
| rs112932561 | 10 | T | C | 0.0347 | 0.018291 | 0.011576 | 0.1141 | 0.069488 | 32.64103 | -0.0783 | 0.082568 | 0.342967 |
| rs1147913 | 10 | T | C | 0.0241 | 0.033969 | 0.014573 | 0.01977 | 0.050345 | 23.17229 | 0.037298 | 0.072099 | 0.604934 |
| rs114836713 | 10 | T | G | 0.0423 | -0.00634 | 0.010527 | 0.5474 | 0.080001 | 38.0089 | -0.0151 | 0.052046 | 0.771754 |
| rs11527805 | 10 | T | C | 0.0582 | 0.035529 | 0.00905 | 8.62E-05 | 0.117699 | 58.30846 | 0.010283 | 0.052412 | 0.844462 |
| rs11595238 | 10 | A | G | 0.9593 | 0.044016 | 0.010722 | 4.04E-05 | 0.085273 | 40.74703 | 0.015691 | 0.09573 | 0.869804 |
| rs117080613 | 10 | T | G | 0.9677 | -0.01422 | 0.011984 | 0.2353 | 0.06076 | 28.27601 | -0.18849 | 0.095859 | 0.049265 |
| rs117239303 | 10 | T | C | 0.016 | -0.06037 | 0.018069 | 0.000836 | 0.027907 | 12.54811 | 0.023692 | 0.154107 | 0.877817 |
| rs117258076 | 10 | T | C | 0.9617 | 0.017785 | 0.01104 | 0.1072 | 0.076334 | 36.1225 | 0.136794 | 0.079909 | 0.08692 |
| rs117374324 | 10 | A | C | 0.9862 | -0.03451 | 0.018346 | 0.05996 | 0.025404 | 11.39337 | -0.02703 | 0.086484 | 0.754628 |
| rs117437067 | 10 | A | G | 0.9872 | 0.0019 | 0.022089 | 0.9316 | 0.025369 | 11.3771 | 0.025386 | 0.190055 | 0.893743 |
| rs117754255 | 10 | T | C | 0.0198 | -0.03904 | 0.016011 | 0.01477 | 0.035901 | 16.27645 | -0.15462 | 0.104799 | 0.140098 |
| rs117775345 | 10 | A | G | 0.0206 | 0.034014 | 0.015704 | 0.03034 | 0.043192 | 19.7312 | 0.070665 | 0.104919 | 0.500614 |
| rs118029903 | 10 | T | C | 0.9852 | 0.008863 | 0.018659 | 0.6346 | 0.029683 | 13.37144 | 0.075317 | 0.198869 | 0.704889 |
| rs118107076 | 10 | T | C | 0.0116 | 0.017628 | 0.022775 | 0.4391 | 0.023754 | 10.6353 | 0.150676 | 0.18015 | 0.402935 |
| rs118126359 | 10 | A | C | 0.02 | -0.01381 | 0.015932 | 0.3861 | 0.038132 | 17.32803 | -0.03301 | 0.119107 | 0.781673 |
| rs118128251 | 10 | A | G | 0.9885 | -0.02612 | 0.019907 | 0.1897 | 0.021578 | 9.639848 | -0.50524 | 0.206301 | 0.014324 |
| rs118130940 | 10 | T | C | 0.021 | 0.057466 | 0.015556 | 0.000221 | 0.046126 | 21.13644 | -0.03503 | 0.076596 | 0.647465 |
| rs11813654 | 10 | T | C | 0.9377 | -0.023 | 0.008766 | 0.008696 | 0.111584 | 54.89891 | -0.01021 | 0.05504 | 0.852808 |
| rs11815839 | 10 | A | G | 0.959 | -0.04322 | 0.010685 | 5.23E-05 | 0.072126 | 33.97659 | -0.02841 | 0.058438 | 0.626853 |
| rs12240751 | 10 | A | G | 0.0194 | -0.02166 | 0.015405 | 0.1596 | 0.036434 | 16.52747 | 0.075262 | 0.173031 | 0.663589 |
| rs12415573 | 10 | T | C | 0.9538 | -0.03783 | 0.010093 | 0.000178 | 0.081709 | 38.89275 | -0.01505 | 0.113807 | 0.894809 |
| rs12415584 | 10 | A | G | 0.9771 | -0.02565 | 0.015412 | 0.09619 | 0.042514 | 19.40767 | -0.02523 | 0.1022 | 0.805047 |
| rs12771280 | 10 | A | G | 0.1678 | 0.017434 | 0.00567 | 0.002106 | 0.289196 | 177.8363 | 0.008526 | 0.043233 | 0.84367 |
| rs137913008 | 10 | A | G | 0.0115 | 0.034088 | 0.023888 | 0.1536 | 0.02434 | 10.90414 | -0.36158 | 0.135001 | 0.007399 |
| rs139500147 | 10 | T | C | 0.9614 | 0.019478 | 0.010998 | 0.07662 | 0.077168 | 36.55058 | 0.029296 | 0.057856 | 0.6126 |
| rs140077890 | 10 | T | C | 0.0289 | 0.015328 | 0.012647 | 0.2254 | 0.057877 | 26.8519 | -0.05724 | 0.0734 | 0.435466 |
| rs140419670 | 10 | T | C | 0.0351 | -0.03113 | 0.011513 | 0.00685 | 0.063647 | 29.71102 | -0.05206 | 0.061493 | 0.397246 |
| rs140650103 | 10 | A | G | 0.9107 | 0.025988 | 0.007429 | 0.000469 | 0.171329 | 90.37005 | 0.035254 | 0.050309 | 0.483454 |
| rs141084162 | 10 | A | G | 0.0165 | 0.004491 | 0.016632 | 0.7869 | 0.032748 | 14.79879 | 0.03957 | 0.244943 | 0.871661 |
| rs142691515 | 10 | T | C | 0.0218 | -0.04141 | 0.015275 | 0.006709 | 0.03926 | 17.86148 | 0.065356 | 0.07626 | 0.391438 |
| rs143213392 | 10 | T | C | 0.0139 | -0.02308 | 0.01823 | 0.2057 | 0.026177 | 11.74943 | 0.097373 | 0.22302 | 0.662392 |
| rs143779814 | 10 | A | G | 0.022 | 0.017275 | 0.014444 | 0.2316 | 0.044545 | 20.37807 | -0.08293 | 0.123524 | 0.502012 |
| rs143917618 | 10 | A | G | 0.9716 | 0.031112 | 0.013428 | 0.02048 | 0.05873 | 27.27235 | 0.091635 | 0.101435 | 0.366324 |
| rs144067061 | 10 | T | C | 0.0121 | 0.011221 | 0.020402 | 0.5826 | 0.02445 | 10.95474 | 0.16122 | 0.100094 | 0.107249 |
| rs144937001 | 10 | A | G | 0.9894 | 0.05136 | 0.021958 | 0.01935 | 0.023244 | 10.40182 | 0.188294 | 0.412879 | 0.648352 |
| rs146572787 | 10 | A | G | 0.9854 | 0.026332 | 0.018596 | 0.1567 | 0.03033 | 13.67163 | 0.23964 | 0.200886 | 0.232903 |
| rs146792072 | 10 | T | C | 0.014 | 0.005118 | 0.020805 | 0.806 | 0.027892 | 12.54131 | -0.14402 | 0.240051 | 0.548524 |
| rs1650145 | 10 | T | C | 0.1913 | 0.016666 | 0.005387 | 0.001974 | 0.319896 | 205.5939 | -0.00385 | 0.03938 | 0.92207 |
| rs17135854 | 10 | T | C | 0.1536 | -0.00753 | 0.005876 | 0.2002 | 0.256129 | 150.5006 | 0.059778 | 0.033572 | 0.074977 |
| rs1764076 | 10 | A | G | 0.9007 | 0.024158 | 0.007084 | 0.000649 | 0.187734 | 101.0233 | 0.147769 | 0.056404 | 0.008798 |
| rs182584748 | 10 | T | C | 0.9807 | 0.052936 | 0.016213 | 0.001095 | 0.042083 | 19.20223 | 0.029853 | 0.161816 | 0.853632 |
| rs1831914 | 10 | A | G | 0.9785 | -0.01256 | 0.014607 | 0.39 | 0.041032 | 18.7021 | 0.063976 | 0.185299 | 0.729899 |
| rs1866158 | 10 | T | G | 0.1898 | 0.011508 | 0.005403 | 0.03321 | 0.314713 | 200.7329 | -0.06582 | 0.037595 | 0.079991 |
| rs186772105 | 10 | T | C | 0.0171 | 0.032387 | 0.017913 | 0.07055 | 0.035865 | 16.25943 | -0.06361 | 0.056157 | 0.257369 |
| rs191837324 | 10 | T | C | 0.9812 | -0.02744 | 0.016423 | 0.09473 | 0.034923 | 15.81698 | -0.03482 | 0.120751 | 0.773081 |
| rs192424837 | 10 | A | G | 0.0137 | -0.05157 | 0.021317 | 0.01558 | 0.024376 | 10.92104 | 0.153432 | 0.08778 | 0.080479 |
| rs193045146 | 10 | A | G | 0.0273 | 0.031723 | 0.01427 | 0.0262 | 0.056588 | 26.21813 | -0.01545 | 0.070219 | 0.825823 |
| rs2488024 | 10 | A | G | 0.4513 | 0.010989 | 0.004258 | 0.009866 | 0.506262 | 448.1832 | -0.00241 | 0.028741 | 0.933178 |
| rs3003509 | 10 | T | C | 0.1511 | -0.02144 | 0.005916 | 0.00029 | 0.24577 | 142.4305 | 0.012841 | 0.039491 | 0.745063 |
| rs34600003 | 10 | A | G | 0.0265 | 0.020974 | 0.013191 | 0.1119 | 0.053806 | 24.85571 | -0.26452 | 0.097708 | 0.006785 |
| rs3894600 | 10 | A | G | 0.4862 | 0.015188 | 0.004239 | 0.00034 | 0.515029 | 464.1866 | -0.01464 | 0.028624 | 0.609014 |
| rs3939062 | 10 | A | G | 0.2112 | 0.017467 | 0.005191 | 0.000766 | 0.345035 | 230.2615 | 0.00765 | 0.033993 | 0.821931 |
| rs41291464 | 10 | A | G | 0.033 | -0.0366 | 0.01186 | 0.00203 | 0.059317 | 27.56212 | 0.076201 | 0.080844 | 0.345901 |
| rs4880221 | 10 | T | C | 0.6947 | 0.013654 | 0.004601 | 0.003002 | 0.435927 | 337.7973 | 0.017978 | 0.030578 | 0.556563 |
| rs4881531 | 10 | A | G | 0.2357 | -0.0118 | 0.004992 | 0.01814 | 0.351891 | 237.3212 | -0.10903 | 0.03481 | 0.001735 |
| rs543328 | 10 | T | C | 0.0303 | 0.0285 | 0.01345 | 0.03413 | 0.062211 | 28.99586 | -0.13331 | 0.076508 | 0.081444 |
| rs59146302 | 10 | T | C | 0.9718 | -0.01244 | 0.012798 | 0.331 | 0.053463 | 24.68823 | 0.10756 | 0.087396 | 0.218425 |
| rs59711523 | 10 | A | G | 0.9727 | 0.020257 | 0.014594 | 0.1652 | 0.055305 | 25.58892 | 0.189367 | 0.198821 | 0.34087 |
| rs61873915 | 10 | T | C | 0.027 | 0.028797 | 0.013072 | 0.02762 | 0.055657 | 25.76123 | 0.044166 | 0.090451 | 0.625351 |
| rs66524564 | 10 | T | C | 0.8562 | -0.01379 | 0.006038 | 0.02237 | 0.239544 | 137.6855 | -0.07177 | 0.040658 | 0.077518 |
| rs7072031 | 10 | A | C | 0.5012 | -0.02106 | 0.005219 | 5.45E-05 | 0.479375 | 402.4646 | -0.03489 | 0.028723 | 0.224478 |
| rs7079658 | 10 | A | G | 0.0385 | 0.01001 | 0.011012 | 0.3632 | 0.075533 | 35.71247 | 0.231605 | 0.116326 | 0.046482 |
| rs7091495 | 10 | T | C | 0.9641 | -0.01918 | 0.011472 | 0.09457 | 0.066617 | 31.19631 | -0.06084 | 0.094947 | 0.521634 |
| rs71483327 | 10 | T | C | 0.0104 | -0.06187 | 0.025514 | 0.01532 | 0.018188 | 8.097129 | -0.00394 | 0.184944 | 0.982988 |
| rs715094 | 10 | T | C | 0.5508 | 0.015913 | 0.004259 | 0.000187 | 0.510841 | 456.4706 | 0.015264 | 0.029802 | 0.608534 |
| rs719805 | 10 | T | C | 0.97 | -0.01617 | 0.01242 | 0.1928 | 0.056348 | 26.1001 | 0.142473 | 0.098278 | 0.147144 |
| rs72813909 | 10 | T | C | 0.0259 | 0.017327 | 0.013339 | 0.1939 | 0.052238 | 24.09134 | -0.0253 | 0.100175 | 0.8006 |
| rs72824816 | 10 | A | G | 0.0713 | 0.023564 | 0.008234 | 0.004213 | 0.138823 | 70.4608 | 0.063859 | 0.060916 | 0.294497 |
| rs72826725 | 10 | T | C | 0.98 | 0.02307 | 0.015932 | 0.1475 | 0.041051 | 18.71139 | -0.10861 | 0.141067 | 0.441367 |
| rs72831956 | 10 | T | G | 0.0169 | -0.01153 | 0.017229 | 0.5032 | 0.032472 | 14.66952 | 0.032031 | 0.203717 | 0.87506 |
| rs73311213 | 10 | T | C | 0.0221 | -0.00731 | 0.01456 | 0.6157 | 0.042596 | 19.44688 | -0.52209 | 0.243862 | 0.03228 |
| rs73343691 | 10 | A | C | 0.8313 | -0.02557 | 0.005658 | 6.18E-06 | 0.266496 | 158.8055 | 0.04189 | 0.03236 | 0.195492 |
| rs74156577 | 10 | T | C | 0.0479 | 0.011052 | 0.009921 | 0.2654 | 0.09325 | 44.95077 | -0.16691 | 0.092688 | 0.071738 |
| rs74341586 | 10 | A | G | 0.0271 | -0.03195 | 0.013737 | 0.02003 | 0.049467 | 22.74698 | 0.094973 | 0.151704 | 0.531286 |
| rs74344109 | 10 | A | G | 0.0166 | -0.0364 | 0.017634 | 0.03904 | 0.030357 | 13.68419 | 0.202148 | 0.099472 | 0.042132 |
| rs74491736 | 10 | A | C | 0.0239 | -0.0185 | 0.013872 | 0.1823 | 0.044962 | 20.57813 | -0.17033 | 0.157208 | 0.278605 |
| rs74542441 | 10 | A | G | 0.9508 | 0.030475 | 0.009796 | 0.001866 | 0.099438 | 48.26342 | 0.021215 | 0.063792 | 0.739467 |
| rs7476438 | 10 | T | C | 0.0239 | -0.05599 | 0.013872 | 5.44E-05 | 0.041715 | 19.02726 | 0.190488 | 0.108767 | 0.079888 |
| rs75028114 | 10 | T | C | 0.961 | -0.00789 | 0.010944 | 0.4709 | 0.073784 | 34.82003 | 0.057998 | 0.085611 | 0.498113 |
| rs76259265 | 10 | A | G | 0.0189 | -0.03543 | 0.01693 | 0.03633 | 0.034548 | 15.64132 | -0.0798 | 0.092505 | 0.38835 |
| rs76396645 | 10 | A | G | 0.979 | 0.029715 | 0.014776 | 0.0443 | 0.043636 | 19.94326 | 0.069555 | 0.102024 | 0.495397 |
| rs76961685 | 10 | T | C | 0.0232 | 0.0152 | 0.014074 | 0.2801 | 0.046723 | 21.42318 | 0.017105 | 0.104828 | 0.870383 |
| rs77177013 | 10 | A | G | 0.0256 | 0.042448 | 0.014597 | 0.003636 | 0.05431 | 25.10179 | 0.152198 | 0.125712 | 0.226015 |
| rs77908423 | 10 | A | G | 0.9705 | -0.03135 | 0.012522 | 0.01227 | 0.053779 | 24.84266 | -0.08316 | 0.130392 | 0.523632 |
| rs78768252 | 10 | A | G | 0.0149 | -0.03137 | 0.017488 | 0.0728 | 0.027571 | 12.39267 | 0.050018 | 0.076339 | 0.512335 |
| rs78854530 | 10 | A | G | 0.0112 | 0.035085 | 0.020234 | 0.08294 | 0.023759 | 10.63778 | -0.12971 | 0.202211 | 0.521224 |
| rs7895609 | 10 | A | G | 0.7542 | 0.014433 | 0.004921 | 0.003362 | 0.381623 | 269.7479 | 0.008722 | 0.035909 | 0.808085 |
| rs79021032 | 10 | T | C | 0.9797 | -0.00822 | 0.015024 | 0.5846 | 0.039127 | 17.79886 | -0.0658 | 0.115111 | 0.567592 |
| rs79146511 | 10 | T | C | 0.9781 | -0.04691 | 0.015943 | 0.00326 | 0.039005 | 17.74072 | 0.014637 | 0.194355 | 0.93997 |
| rs79334727 | 10 | A | C | 0.0541 | 0.020287 | 0.009366 | 0.03035 | 0.106584 | 52.14546 | -0.0629 | 0.070612 | 0.373051 |
| rs79658539 | 10 | T | C | 0.0133 | -0.05378 | 0.019693 | 0.006309 | 0.02357 | 10.55089 | -0.01027 | 0.094343 | 0.913288 |
| rs79692371 | 10 | T | C | 0.0377 | -0.0329 | 0.011124 | 0.003096 | 0.067936 | 31.85911 | 0.151203 | 0.075031 | 0.043884 |
| rs876414 | 10 | T | C | 0.3162 | 0.014011 | 0.004556 | 0.002103 | 0.444724 | 350.0733 | 0.012194 | 0.030041 | 0.684811 |
| rs9329312 | 10 | A | G | 0.8844 | 0.01526 | 0.006626 | 0.02128 | 0.21081 | 116.7581 | 0.038093 | 0.045422 | 0.401661 |
| rs9971328 | 10 | A | G | 0.856 | -0.02125 | 0.006035 | 0.000428 | 0.236268 | 135.2201 | 0.055653 | 0.036997 | 0.132516 |

**SNP, single nucleoid polymorphism; CHR, chromosome; EA, effect allele. OA, other allele. EAF, effect allele frequency. SE, standard error. R^2^, explained phenotypic variability; F, F statistic; Beta, effect estimate; SE, standard error.**

**Table S2 6 SNPs used as genetic instruments for the causality between sjogren’s syndrome and periodontitis**

| SNP | CHR | EA | OA | EAF | Exposure-Sjogren’s syndrome | | | R2 | F | Outcome-Periodontitis | | |
| --- | --- | --- | --- | --- | --- | --- | --- | --- | --- | --- | --- | --- |
|  |  |  |  |  | Beta | SE | P-value |  |  | Beta | SE | P-value |
| rs11007806 | 10 | A | G | 0.027571 | -0.43449 | 0.099568 | 1.28E-05 | 0.010123 | 3763.528 | 0.000702 | 0.008064 | 0.9305 |
| rs11017904 | 10 | A | C | 0.21794 | -0.14673 | 0.03533 | 3.28E-05 | 0.007339 | 2720.962 | 0.010415 | 0.005704 | 0.0678 |
| rs11250633 | 1 | T | C | 0.323147 | 0.152319 | 0.029802 | 3.20E-07 | 0.010149 | 3773.474 | 0.007432 | 0.004502 | 0.09877 |
| rs12761735 | 10 | C | T | 0.07759 | 0.218671 | 0.050091 | 1.27E-05 | 0.006845 | 2536.328 | -0.00138 | 0.008036 | 0.8635 |
| rs2688611 | 10 | T | G | 0.648419 | -0.13344 | 0.02956 | 6.35E-06 | 0.008119 | 3012.417 | -0.0011 | 0.004808 | 0.8193 |
| rs7914671 | 4 | T | C | 0.2133 | 0.149749 | 0.03362 | 8.42E-06 | 0.007526 | 2790.726 | 0.008445 | 0.005369 | 0.1157 |

**SNP, single nucleoid polymorphism; CHR, chromosome; EA, effect allele. OA, other allele. EAF, effect allele frequency. SE, standard error. R^2^, explained phenotypic variability; F, F statistic; Beta, effect estimate; SE, standard error.**

**Figure S1 MR leave-one-out sensitivity analysis for periodontitis on Sjögren’s syndrome**


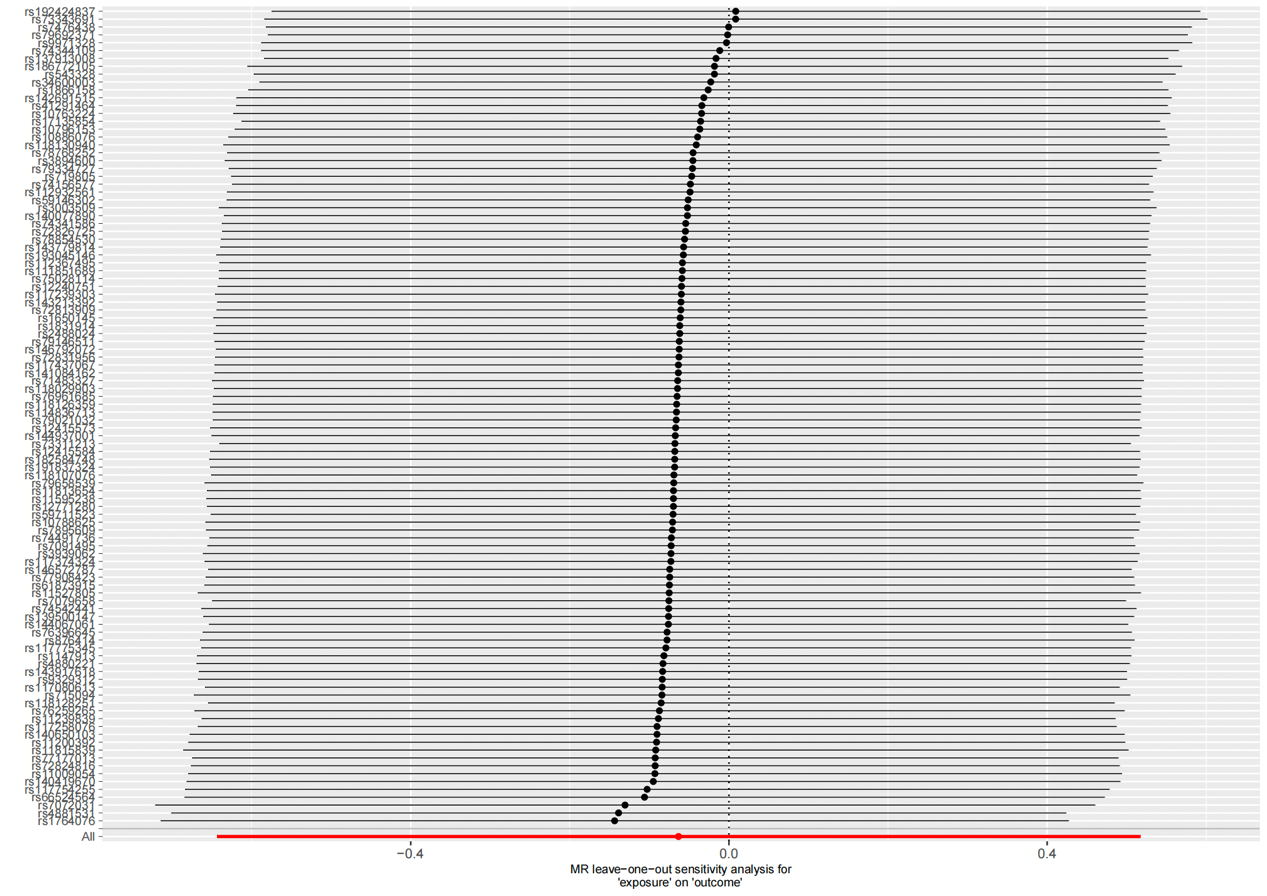


**Figure S2 MR leave-one-out sensitivity analysis for Sjögren’s syndrome on periodontitis**


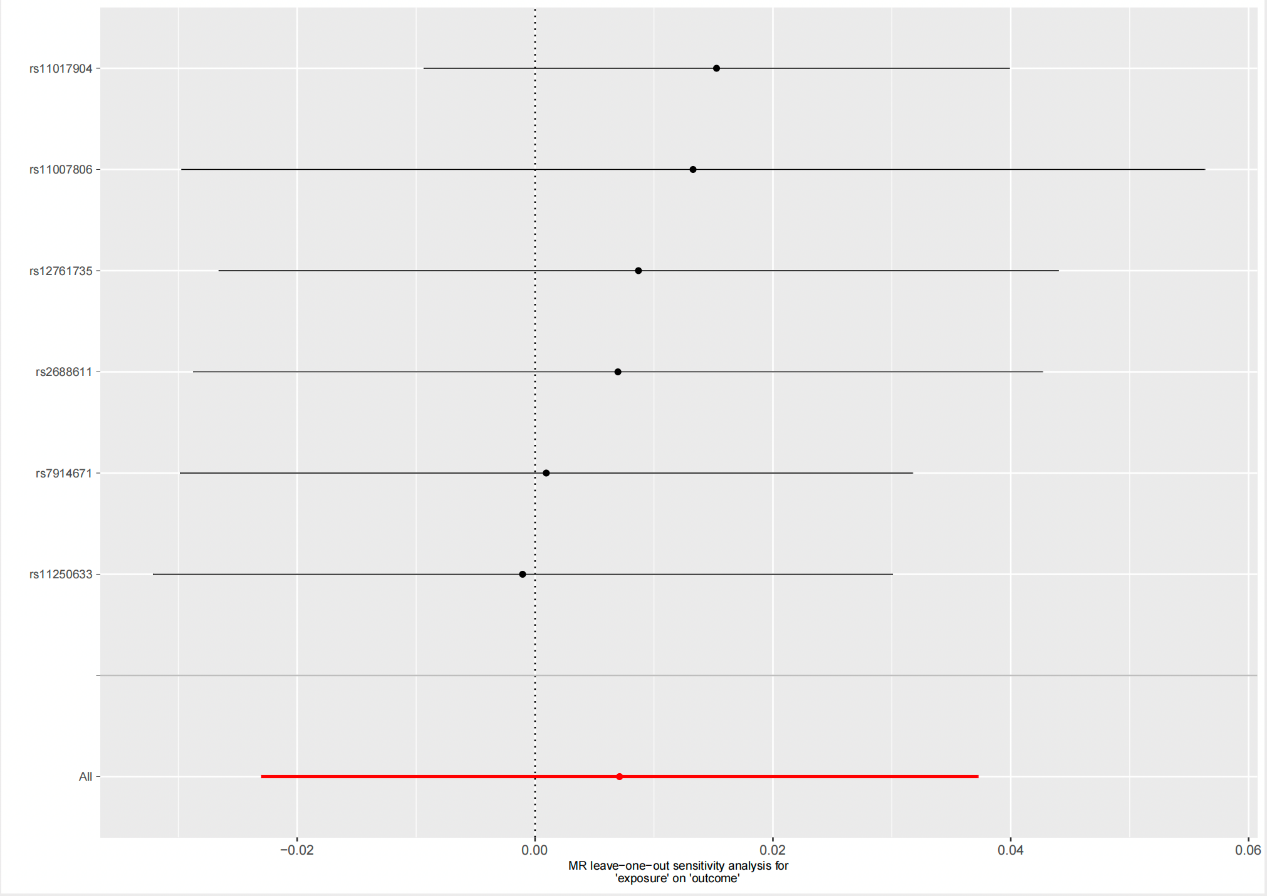


**Figure S3 Funnel plot of SNPs associated with periodontitis**


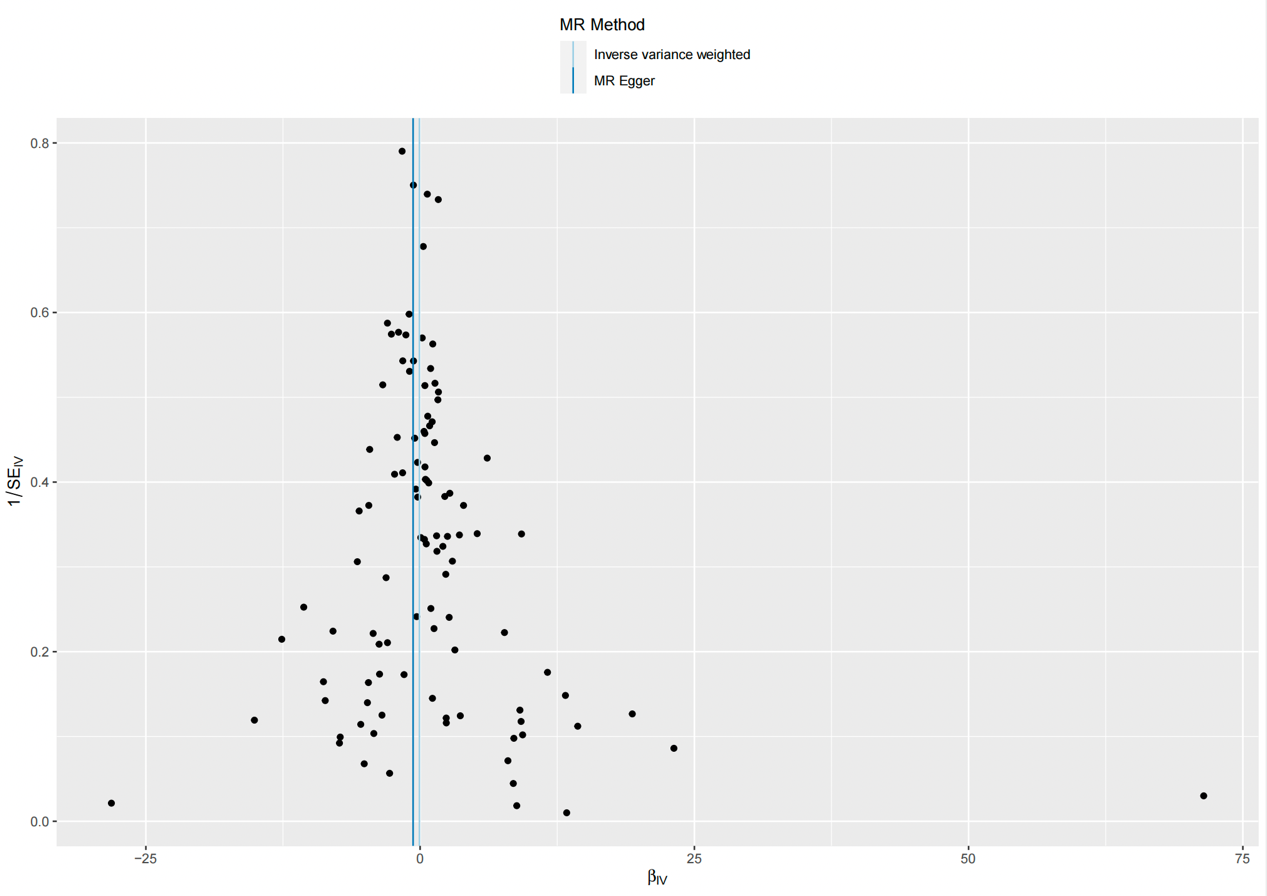


**Figure S4 Funnel plot of SNPs associated with Sjögren’s syndrome**


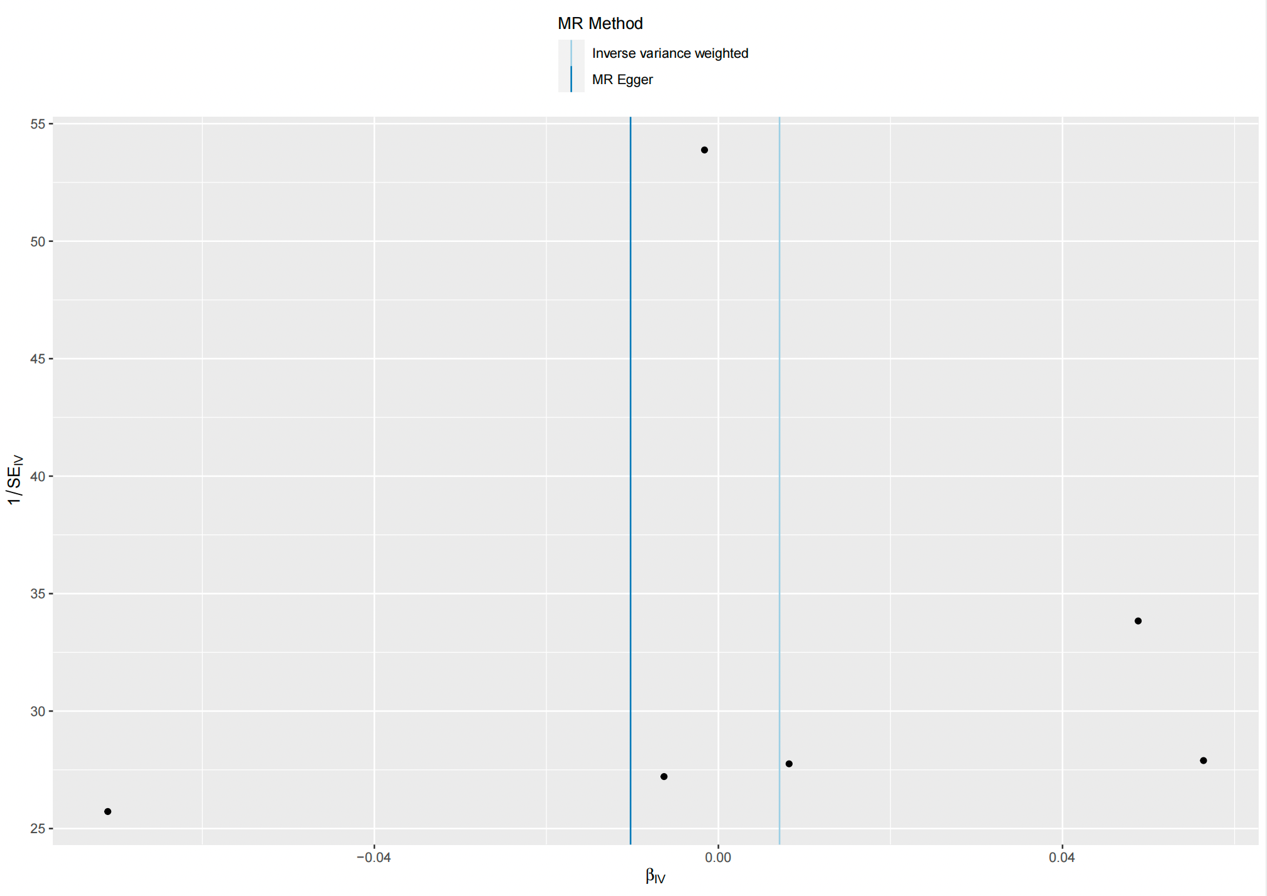

Supplement: Supplementary file 1 — Supplementary Material 1 [file 12903_2024_4151_MOESM1_ESM.docx]
